# Efficient derivation of sympathetic neurons from human pluripotent stem cells with a defined condition

**DOI:** 10.1038/s41598-018-31256-1

**Published:** 2018-08-27

**Authors:** Kosuke Kirino, Tatsutoshi Nakahata, Tomoaki Taguchi, Megumu K. Saito

**Affiliations:** 1 Department of Clinical Application, Center for iPS Cell Research and Application (CiRA), Kyoto University, Kyoto, 606-8507 Japan; 2 Department of Pediatric Surgery, Graduate School of Medical Science, Kyushu University, Fukuoka, 812-8582 Japan

## Abstract

Sympathetic neurons (SNs) are an essential component of the autonomic nervous system. They control vital bodily functions and are responsible for various autonomic disorders. However, obtaining SNs from living humans for *in vitro* study has not been accomplished. Although human pluripotent stem cell (hPSC)-derived SNs could be useful for elucidating the pathophysiology of human autonomic neurons, the differentiation efficiency remains low and reporter-based cell sorting is usually required for the subsequent pathophysiological analysis. To improve the efficiency, we refined each differentiation stage using PHOX2B::eGFP reporter hPSC lines to establish a robust and efficient protocol to derive functional SNs via neuromesodermal progenitor-like cells and trunk neural crest cells. Sympathetic neuronal progenitors could be expanded and stocked during differentiation. Our protocol can selectively enrich sympathetic lineage-committed cells at high-purity (≈80%) from reporter-free hPSC lines. Our system provides a platform for diverse applications, such as developmental studies and the modeling of SN-associated diseases.

## Introduction

The differentiation of human pluripotent stem cells (hPSCs) into specific cell populations is a major avenue for developmental studies, disease modeling and regenerative medicine^1,2^. Various neuronal subtypes, such as somatic motor neurons, midbrain dopaminergic neurons and peripheral sensory neurons, have been differentiated from hPSCs efficiently^3–5^. In contrast, sympathetic neurons (SNs), which are one of the two main divisions of the autonomic nervous system (ANS), have not. Although several previous reports have described differentiation protocols for SNs^6–9^, the differentiation efficiency remains relatively low. This inefficiency is mainly because sympathetic lineage-committed progenitor cells^10^ were not fully detected or characterized during the differentiation.

Accumulating evidence indicates that SNs are derived from trunk neural crest cells (NCCs)^11^, which arise from neuromesodermal progenitor cells (NMPs)^12,13^. NMPs are bipotent for the caudal neural plate and paraxial mesoderm during the axial development of embryos. Committed sympathetic neuronal progenitor cells express *PHOX2B*, an essential transcription factor required for sympathetic neuronal differentiation^14^. *PHOX2B* is expressed throughout the course of sympathetic neuronal differentiation, including in post-mitotic neurons^15^.

By translating the *in vivo* and *ex vivo* findings of animal experiments, the *in vitro* derivation of NMP-like cells from hPSCs has been reported^16–18^. Canonical WNT signaling was shown to play an essential role in the specification of human NMPs. With the coordination of WNT signaling, bone morphogenic proteins (BMPs) dorsalize NMP-like cells and encourage their development into NCCs^17,19,20^. However, subsequent *in vitro* developmental pathways towards SNs have not been described quantitatively. In the present study, using PHOX2B::eGFP reporter hPSC lines, we optimized the culture conditions that selectively expand sympathetic neural crest-derived cells and encourage their development into SNs with high efficiency.

## Results

### Induction of *PHOX2B*-expressing NCCs via NMP-like cells using PHOX2B::eGFP reporter hPSC lines

There is no specific surface marker for the detection of sympathetic neuronal lineage cells. To address this issue, we conducted reporter-based tracking of *PHOX2B* expression during differentiation. Given the long-sustained *PHOX2B* expression from the progenitor stage to the mature neuron stage^15^, we hypothesized that tracking *PHOX2B* expression can help optimize the differentiation protocol. We targeted the 3′UTR region of the *PHOX2B* locus and generated PHOX2B::eGFP knocked-in reporter lines from two hPSC clones: human embryonic stem cells (hESCs, cell line: KhES1) and human induced pluripotent stem cells (hiPSCs, cell line: 409B2) (Supplementary Fig. 1a–c).

SNs are derived from trunk NCCs^11^, which originate from NMPs^12,13^. For NMP induction, WNT-mediated caudalization of hPSCs is essential^16–18^. We first evaluated the effect of a WNT activator, CHIR99021, during the first 3 days of aggregation culture of hPSCs. Treatment with 1.5 µM or more CHIR was effective for the upregulation of *HOX* genes, indicating that cells in these conditions began to be caudalized over the initial 3 days of differentiation (Supplementary Fig. 1d). *BRACHURY* and *SOX2*, two markers for NMPs^12,13^, were also expressed under these conditions, confirming that CHIR-treated day 3 aggregates had characteristics of NMPs. As previously reported^21^, treatment with a higher dosage of CHIR (5 µM) is more likely to direct hPSCs to a mesodermal fate, based on the up-regulation of *TBX6*, a mesoderm-specific transcription factor^13^.

**Figure 1:**
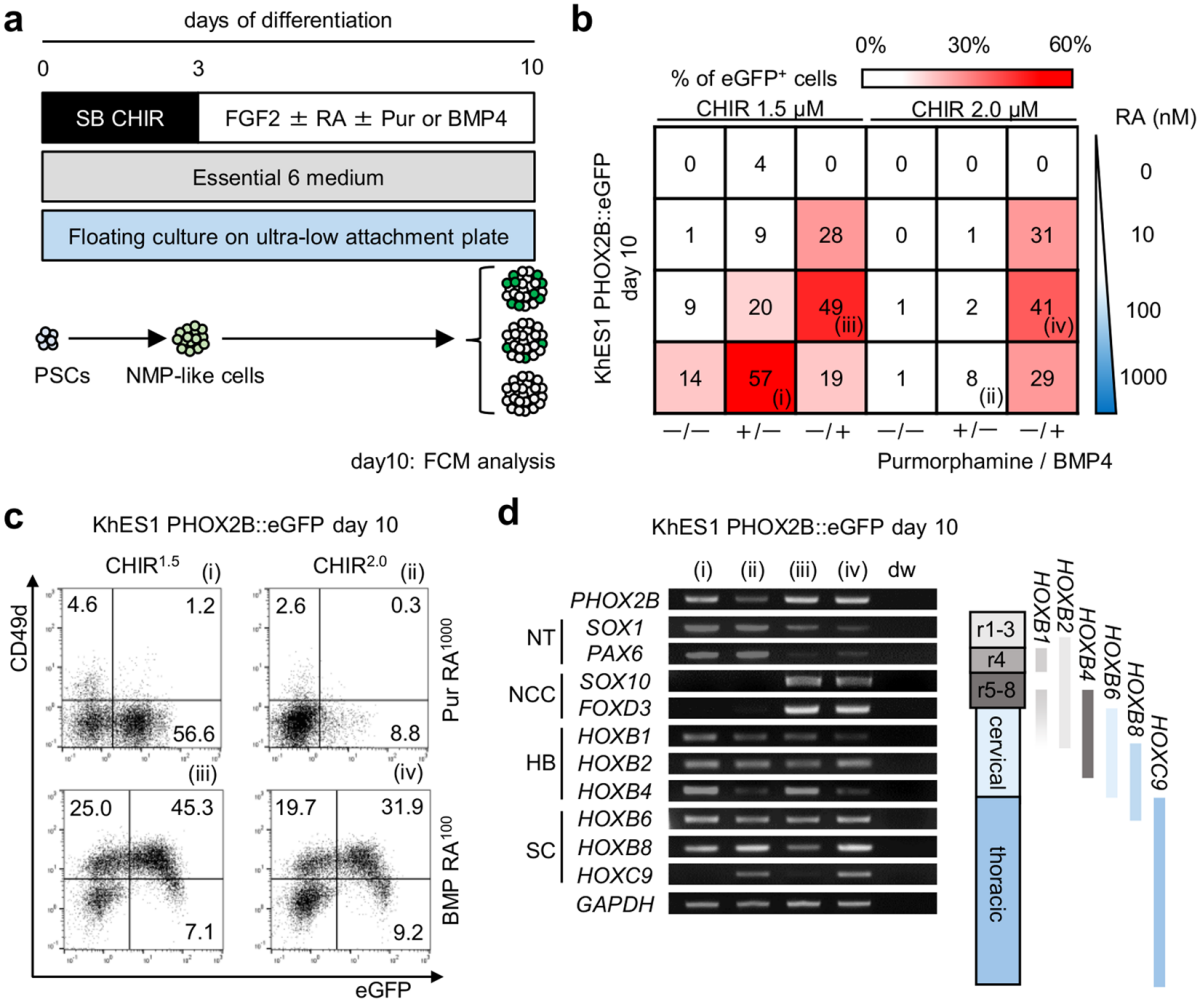
Modulation of rostro-caudal and dorso-ventral axis specification induces the development of *PHOX2B*-expressing NCCs from hPSCs. (**a**) A diagram of the culture conditions for the modification of rostro-caudal and dorso-ventral axis specification of hPSCs. (**b**) A heat map image showing the percentage of eGFP^+^ cells on day 10 of differentiation using KhES1 PHOX2B::eGFP under various conditions. (**c**) Representative FCM plots of day 10 KhES1 PHOX2B::eGFP-derived aggregates under the indicated conditions (i–iv) of (**b**). (**d**) RT-PCR analyses for *PHOX2B*, *SOX1*, *PAX6*, *SOX10*, *FOXD3*, *HOXB1*, *HOXB2*, *HOXB4*, *HOXB6*, *HOXB8* and *HOXC9* in day 10 aggregates under conditions (i–iv). The right diagram shows the expression pattern of *HOX* genes in the rhombomere (r1–8) and the spinal cord (cervical and thoracic) region. SB = SB431542, CHIR = CHIR 99021, RA = retinoic acid, Pur = Purmorphamine, BMP = BMP4, NT = neural tube, NCC = neural crest cell, HB = hindbrain, SC = spinal cord.

In the caudal region of the body, various subtypes of neural progenitor cells (NPCs) and NCCs are generated from NMP-derived neural plates through dorso-ventral specification^19,20^. We therefore modified the dorso-ventral axis of the day 3 aggregates. Since BMPs and sonic hedgehog (SHH) signals are important for dorsalization and ventralization, respectively^20^, we added BMP4 and the SHH agonist Purmorphamine (Pur) to 1.5, 2.0 or 3.0 µM CHIR-treated day 3 aggregates (Fig. 1a). In addition, we used retinoic acid (RA), because RA is effective for NPC induction via NMPs^20^ and because RA signaling controls the initiation of trunk NCC emigration^22^. Indeed, neuronal induction of primary trunk NCCs from neural tube explants has been previously performed in the presence of RA^23^. In several conditions, we detected PHOX2B::eGFP^+^ cells at over 40% purity using flow cytometry (FCM) analyses (Fig. 1b and Supplementary Fig. 1e). Among these cells, we focused on the following four conditions: (i) CHIR^1.5 μM^Pur^+^RA^1000 *n*M^, (ii) CHIR^2.0 μM^Pur^+^RA^1000 *n*M^, (iii) CHIR^1.5 μM^BMP^+^RA^100 *n*M^ and (iv) CHIR^2.0 μM^BMP^+^RA^100 *n*M^. With 3.0 µM CHIR treatment, a relatively lower frequency of eGFP^+^ cells was detected (0%–10%) compared to 1.5 or 2.0 µM CHIR treatment (data not shown).

*PHOX2B* is expressed not only in autonomic neural crest derivatives, but also in central nervous system (CNS) neurons and their progenitor cells in the hindbrain^15,24^. Since CD49d (Integrin alpaha-4) is expressed in migratory NCCs and their derivatives^25,26^, we used it to distinguish NCCs from other lineages, such as NPCs, in the CNS. We detected more CD49d^+^ cells under BMP-treated conditions (conditions (iii) and (iv)) than under Pur-treated conditions (conditions (i) and (ii)) (Fig. 1c). Only BMP4-treated cells (conditions (iii) and (iv)) expressed the NCC markers *SOX10* and *FOXD3*, whereas the NPC markers *SOX1* and *PAX6* were more strongly expressed in Pur-treated cells (Fig. 1d). Interestingly, in our experiments, RA was essential for the upregulation of *PHOX2B* under BMP4-treated conditions (Fig. 1b and Supplementary Fig. 1e,i). Although SOX10^+^ cells were detected both with and without RA, SOX10^+^ and PHOX2B^+^ double-positive cells were found only under conditions with RA (Supplementary Fig. 1j,k), indicating that BMP4 plays a role in NCC induction and that RA modifies the fate of NCCs towards autonomic lineages in our differentiation culture.

Some previous reports have described that the dosage of the WNT signal determines the level of rostro-caudal axis specification^27,28^. In agreement with this finding, we found that higher CHIR concentration corresponded with more specificity in caudal cells. CHIR treatment at 1.5 µM assigned cells to the hindbrain and the cervical spinal cord region (*HOXB4*^+^*HOXB8*^+^*HOXC9*^−^), whereas 2.0 µM CHIR treatment assigned cells to the cervical to thoracic spinal cord region (*HOXB4*^−^*HOXB8*^+^*HOXC9*^+^)^29^ (Fig. 1d).

Under Pur-treated conditions, most eGFP^+^ cells did not express CD49d (Fig. 1c and Supplementary Fig. 1f). Since SHH signal ventralizes neuroepithelial cells, we hypothesized the eGFP^+^ cells under conditions (i,ii) are progenitor cells of cranial motor neurons in the ventral hindbrain^3,24^. Indeed, these CD49d^−^eGFP^+^ cells differentiated into neurons which expressed the motor neuron marker choline acetyl transferase (ChAT) (Supplementary Fig. 1g,h)^3^.

Based on the above findings, we thus confirmed that 1) CHIR-treated day 3 aggregates can give rise to both CNS neural progenitors and NCCs; 2) BMP and RA treatment are essential for the induction of *PHOX2B*-expressing NCCs; and 3) 2.0 µM CHIR treatment leads hPSCs to the trunk level of rostro-caudal axis specification. We found that eGFP^+^ cells expressed PHOX2B protein under these conditions, excluding the possibility of leakage of our reporter system (Supplementary Fig. 1l).

### Purified *PHOX2B*-expressing NCCs showed characteristics of embryonic sympathetic ganglion progenitor cells

**Figure 2:**
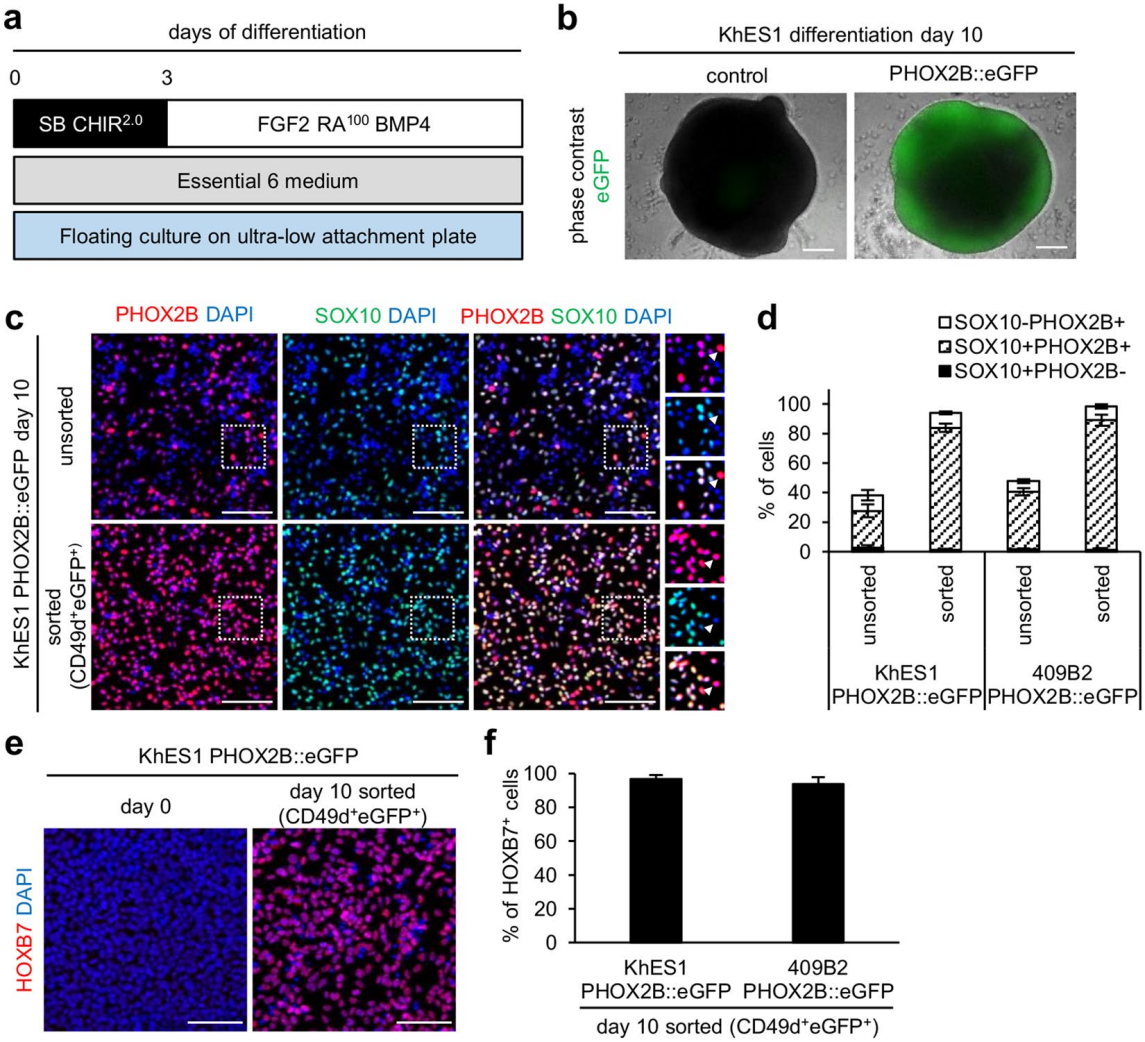
SOX10 and PHOX2B, essential transcription factors of sympathetic NCCs, are expressed in CD49d^+^eGFP^+^ cells. (**a**) A diagram of the culture condition for sympathetic NCC differentiation. (**b**) Representative images of day 10 aggregates of KhES1 control and KhES1 PHOX2B::eGFP (scale bars = 200 µm). (**c**) Immunocytochemistry analyses of unsorted and CD49d^+^eGFP^+^-sorted day 10 differentiated cells for PHOX2B and SOX10 (scale bars = 50 µm). The white dashed boxes mark the areas enlarged in the right panels. The white arrowheads indicate PHOX2B^+^SOX10^−^ cells. (**d**) Quantification of PHOX2B^+^ and SOX10^+^ cells in (**c**) (mean ± s.d., n = 3). (**e**) Immunocytochemistry analyses for HOXB7 on day 0 and day 10 (CD49d^+^eGFP^+^-sorted) of differentiation (scale bars = 50 µm). (**f**) Quantification of HOXB7^+^ cells in (**e**) (mean ± s.d., n = 3). SB = SB431542, CHIR = CHIR 99021, RA = retinoic acid.

To confirm the derivation of sympathetic neuronal lineage cells in our culture, we next characterized CD49d^+^eGFP^+^ cells under CHIR^2.0 μM^BMP^+^RA^100 nM^ condition (Fig. 2a,b). During the differentiation, CD49d^+^eGFP^+^ cells emerged after day 8, and some eGFP^+^ cells lost their CD49d expression after day 10 (Supplementary Fig. 2a,b), suggesting the coexistence of cells in different stages. As expected, among eGFP^+^ cells, most CD49d^+^ cells expressed the neuronal marker TUBBIII weakly, whereas CD49d^−^ cells expressed TUBBIII strongly (Supplementary Fig. 2c,d), indicating that CD49d^−^eGFP^+^ cells are in a later stage of neuronal commitment. Most CD49d^+^eGFP^+^ cells were double-positive for SOX10 and PHOX2B (Fig. 2c,d and Supplementary Fig. 2e), which are essential transcription factors for autonomic NCCs. They also expressed HOXB7 (Fig. 2e,f and Supplementary Fig. 2f), a marker for the trunk (spinal cord) level of the rostro-caudal axis^29^. These findings indicate that the cells are autonomic NCCs at the trunk level. Considering that trunk NCCs give rise to SNs but not other autonomic neuronal lineages, such as parasympathetic and enteric neurons, the expression of essential transcription factors in CD49d^+^eGFP^+^ cells under CHIR^2.0 μM^BMP^+^RA^100 nM^ condition is compatible with that in murine sympathetic ganglion progenitor cells *in vivo* and *ex vivo*^30,31^.

### Maintenance of *PHOX2B* expression causes sympathetic neuronal progenitor cells to commit to SNs

**Figure 3:**
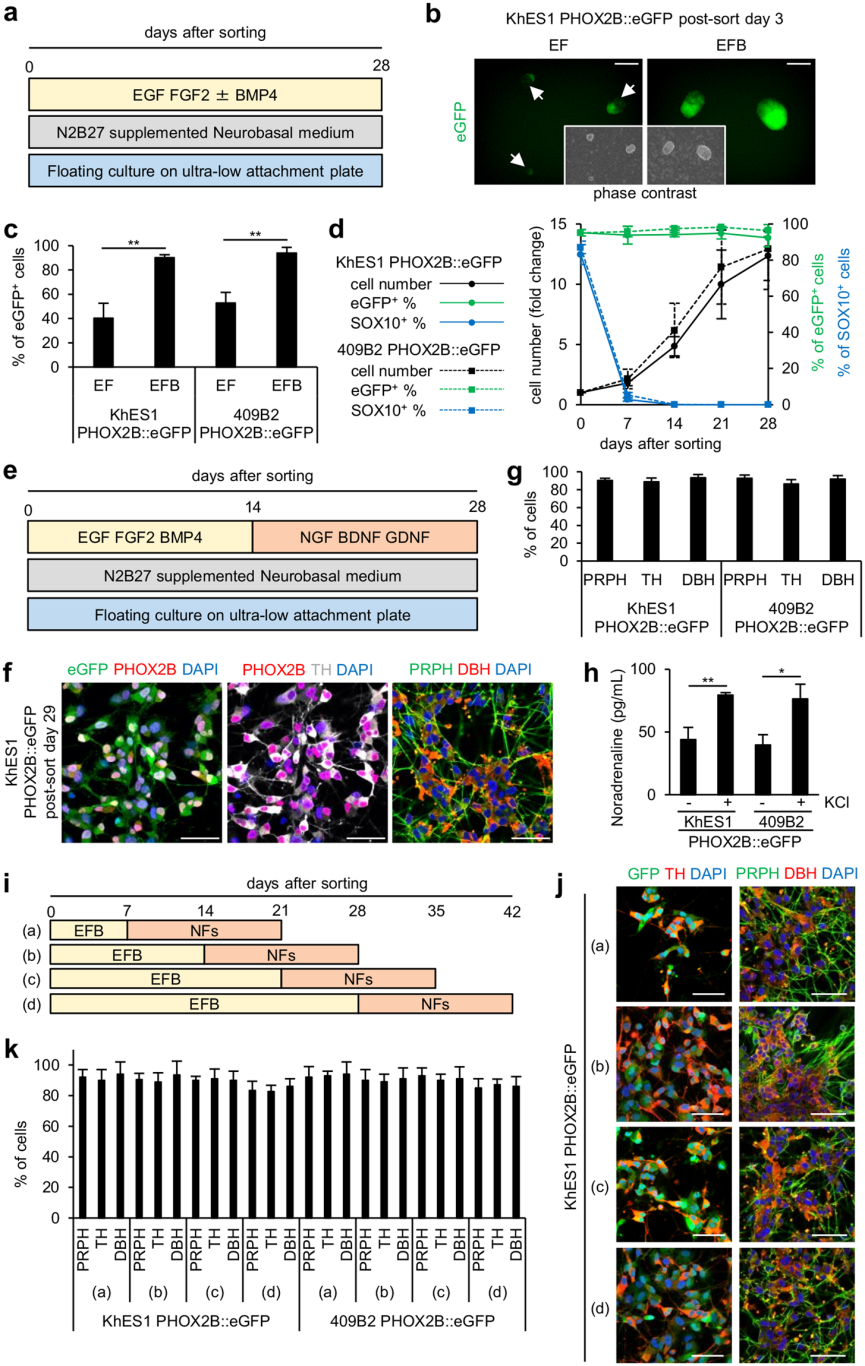
Maintenance of *PHOX2B* expression causes sympathetic NCCs to commit to SNs. (**a**) A diagram of the culture conditions for sympathetic NCC culture after FACS purification. (**b**) Representative images of post-sorting day 3 neurospheres under conditions of EF and EFB (scale bars = 200 µm). (**c**) Quantitation of eGFP^+^ cells among post-sorting day 7 neurospheres under conditions of EF and EFB by FCM analyses (mean ± s.d., n = 3, **P < 0.01; Student’s t-test). (**d**) Transition of cell numbers and percentage of eGFP^+^ cells and SOX10^+^ cells during 28-day culture under EFB conditions after sorting (mean ± s.d., n = 3). Cell numbers are described as the fold-change over the numbers on day 0. (**e**) A diagram of the culture conditions for sympathetic neuronal differentiation after FACS purification. (**f**) Immunocytochemistry analyses for eGFP, PHOX2B, TH, PRPH and DBH on day 29 after sorting (scale bars = 50 µm). (**g**) Quantification of PRPH^+^, TH^+^ and DBH^+^ cells in (**f**) (mean ± s.d., n = 3). (**h**) Quantification of noradrenaline concentrations in the supernatant of cultured SNs on day 30 after sorting with (+) or without (−) KCl treatment (mean ± s.d., n = 3, ^*^P < 0.05, ^**^P < 0.01; Student’s t-test). (**i**) A diagram of the culture conditions for the neuronal induction of neurosphere culture. (**j**) Immunocytochemistry analyses for eGFP, TH, PRPH and DBH under various neuronal induction conditions in (**i**) (scale bars = 50 μm). (**k**) Quantification of PRPH^+^, TH^+^ and DBH^+^ cells in (**j**) (mean ± s.d., n = 3). EF = EGF + FGF2; EFB = EGF + FGF2 + BMP4; NFs = neurotrophic factors: NGF, BDNF and GDNF.

We next optimized the culture conditions of CD49d^+^eGFP^+^ sympathetic neuronal progenitor cells for the induction of SNs. Because SNs maintain the expression of *PHOX2B* throughout differentiation, whereas non-neuronal cells derived from sympathetic ganglion progenitor cells lose their *PHOX2B* expression^31,32^, the eGFP expression was again tracked. Additionally, since sympathetic ganglion progenitor cells can be selectively expanded *in vitro* by a neurosphere culture method^30,31^, we cultured sorted CD49d^+^eGFP^+^ cells in suspension with EGF and FGF2 to form aggregates (Fig. 3a). However, about half of the cells lost eGFP expression during the first seven days of culture after sorting (Fig. 3b,c and Supplementary Fig. 3a). In animal experiments *in vivo* and *ex vivo*, BMP4 is required for the initial neuronal commitment of sympathetic ganglion progenitor cells^33,34^. We therefore added BMP4, which maintained the expression of eGFP in most of the cells (>90%) (Fig. 3b,c and Supplementary Fig. 3a). Furthermore, when we cultured the cells longer (until 28 days post-sorting), this condition expanded the cell number more than 10-fold without loss of eGFP expression (Fig. 3d). Finally, we confirmed that the expanded cells were in the proliferating phase of the cell cycle because around 40% of cells expressed KI-67 (Supplementary Fig. 3b,c).

During the prolonged aggregation culture, the expression of SOX10 rapidly decreased, and almost no cells expressed SOX10 after 14 days (Fig. 3d and Supplementary Fig. 3d). Considering that the maintenance of *PHOX2B* together with the loss of *SOX10* guides sympathetic ganglion progenitor cells to neuronal commitment^31,32^, neurosphere culture with BMP4 seemed to encourage the development of these progenitors into neurons. Indeed, with BMP4, the numbers of TUBBIII^+^ cells increased during the first 7 days of culture (Supplementary Fig. 3e,f). In adhesion culture, even in the presence of BMP4, most cells lost their eGFP expression, and some expressed alpha smooth muscle actin, a marker for neural crest-derived myofibroblasts^30^ (Supplementary Fig. 3g), indicating the importance of floating aggregate culture for specification into neurons. Overall, we successfully established a culture system which propagates sympathetic neuronal lineage cells while maintaining *PHOX2B* expression.

We next derived SNs from the neurosphere culture and evaluated their purity. The plated BMP4-treated aggregates did not show neuronal morphology (Supplementary Fig. 3h), probably owing to their immaturity. For the maturation of neuronal progenitors, we removed EGF, FGF2 and BMP4, all of which are reported to be involved in the initial neuronal commitment of SNs but are not sufficient for inducing mature neurons^35,36^, and added the neurotrophic factors (NFs) NGF, BDNF and GDNF, which promote sympathetic neuronal development^37–39^ (Fig. 3e). After 14-day culture with this modified protocol, the cells showed axonal morphology (Supplementary Fig. 3h). At the time, most of the cells (over 85%) were positive for tyrosine hydroxylase (TH) and dopamine beta hydroxylase (DBH), both of which are catalytic enzymes of catecholamine synthesis and thus markers for noradrenergic neurons^10^ (Fig. 3f,g). These cells also expressed peripherin (PRPH), which is a peripheral neuron-specific intermediate filament^40^. Thus, the expression pattern of these cells was compatible with that of SNs, which were peripheral (PRPH^+^) noradrenergic (TH^+^DBH^+^) neurons derived from trunk NCCs.

Finally, to test the functionality of these neurons, we confirmed the release of noradrenaline (NA), a neurotransmitter of SNs (Fig. 3h). Treatment with potassium chloride (KCl) significantly increased the concentration of NA, indicating that KCl-mediated membrane depolarization evoked NA release. The amount of released NA from hPSC-derived SNs was comparable to that in a previous report^8^.

The maturation step was applicable to neurosphere cells at different time points of post-sorting culture (Fig. 3i). Therefore, most *PHOX2B-*expressing neurosphere cells gave rise to terminally differentiated neurons even through prolonged (28 days) culture (Fig. 3j,k). Furthermore, neurosphere cells can be freeze-stocked by a common method without losing viability or neuronal differentiation potential (Supplementary Fig. 3i–m).

### Derivation of highly enriched SNs without cell sorting

Through refinement of the culture conditions, we successfully differentiated hPSCs into SNs in a step-wise manner. However, such differentiation was only possible with PHOX2B::eGFP reporter hPSC lines, as a cell sorting step is necessary for purifying *PHOX2B*-expressing NCCs. Since our ultimate goal is to develop a robust and universal differentiation method applicable to various hPSC lines, we next tried to apply our system to hPSC lines without *PHOX2B* reporter.

**Figure 4:**
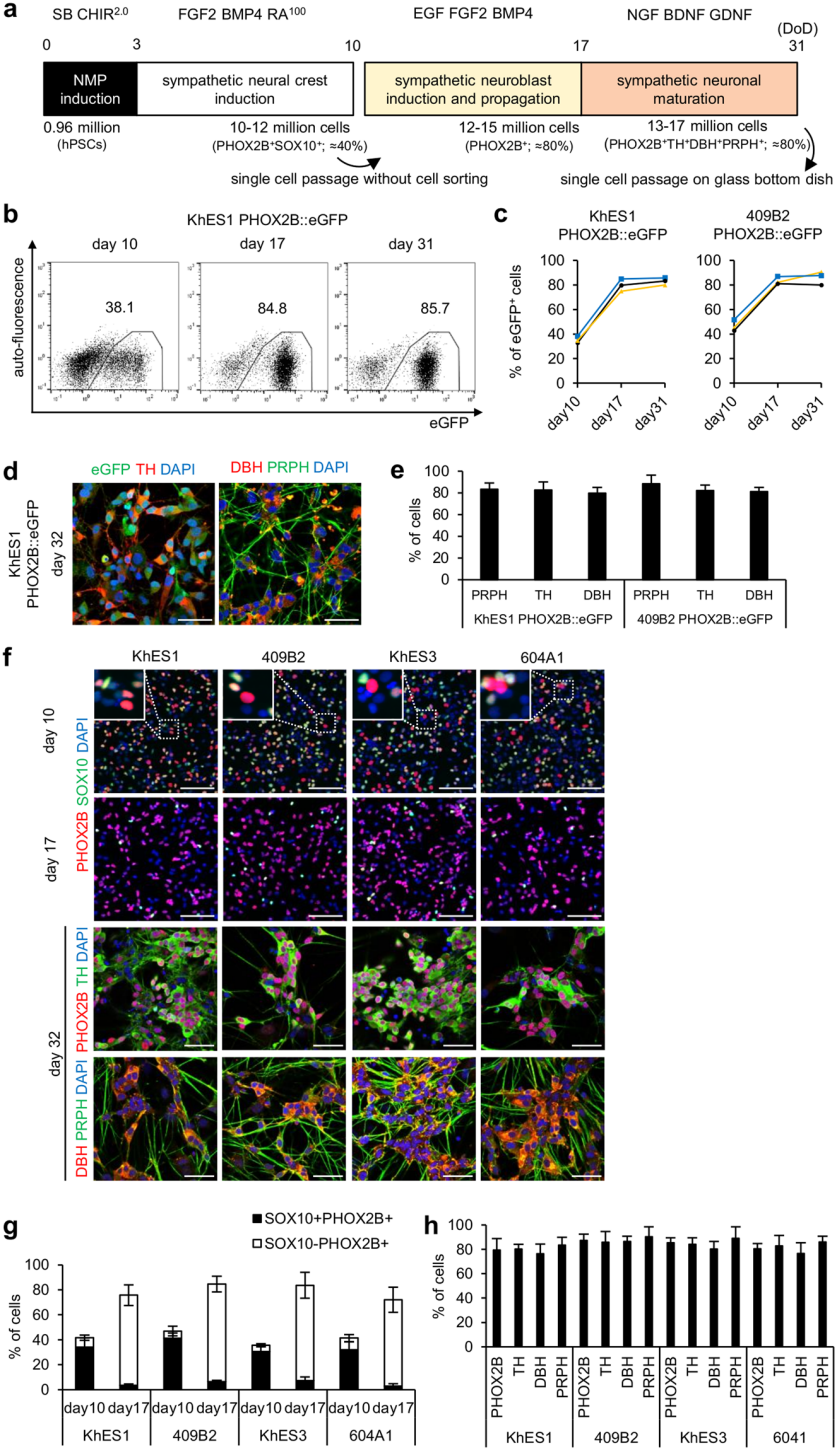
Derivation of highly enriched SNs without cell sorting. (**a**) A diagram of the culture conditions for sympathetic neuronal differentiation without cell sorting. (**b**) FCM analyses of eGFP^+^ cells on days 10, 17 and 31 of the differentiation shown in (**a**). (**c**) Quantification of eGFP^+^ cells in (**b**). Data from three independent experiments are shown in each graph. (**d**) Immunocytochemistry analyses for eGFP, TH, PRPH and DBH on day 32 of the differentiation (scale bars = 50 µm). (**e**) Quantification of PRPH^+^, TH^+^ and DBH^+^ cells in (**d**) (mean ± s.d., n = 3). (**f**) Immunocytochemistry analyses for PHOX2B and SOX10 on days 10 and 17, and for TH, DBH and PRPH on day 32 (scale bars = 50 µm). The white dashed boxes in the day 10 images mark the areas enlarged in the top left of the same panels. (**g**) Quantification of PHOX2B^+^ and SOX10^+^ cells on days 10 and 17 in (**f**) (mean ± s.d., n = 3). (**h**) Quantification of PHOX2B^+^, TH^+^, DBH^+^ and PRPH^+^ cells on day 32 in (**f**) (mean ± s.d., n = 3). SB = SB431542, CHIR = CHIR 99021, RA = retinoic acid, DoD = days of differentiation.

To this end, we repeated the same procedure as described above except for cell sorting using PHOX2B::eGFP hPSC lines (Fig. 4a). A time course analysis surprisingly showed that eGFP^+^ cells were highly purified (day 17; 75–85%) after transfer to neurosphere culture and highly maintained (day 31; 75–90%) in neurophere cells after the neuronal maturation step (Fig. 4b,c). Immunostaining proved PHOX2B^+^TH^+^DBH^+^PRPH^+^ peripheral noradrenergic neuronal characteristics in 75–80% of the cells (Fig. 4d,e), indicating that the current protocol can selectively expand sympathetic neuronal lineage cells without cell sorting.

As further proof of this principle, we performed the same procedure using four different hPSC lines without PHOX2B::eGFP reporter (KhES1 and KhES3 as hESC lines, and 409B2 and 604A1 as hiPSC lines). In this series of experiments, PHOX2B^+^TH^+^DBH^+^PRPH^+^ SNs were robustly generated at 70–80% purity through neurosphere culture (Fig. 4f–h). We confirmed NA release from these SNs (Supplementary Fig. 4). These data prove the robustness of our protocol for generating SNs from various hPSC lines.

## Discussion

SNs are one of the two main divisions of the ANS, along with parasympathetic neurons. Both SNs and parasympathetic neurons are neural crest derivatives.

Although the SNs of chicken and murine have been well characterized, there are few reports of hPSC-derived SNs. This is because (1) there is no specific surface marker for detecting sympathetic neural lineage cells, and (2) cranial NCCs can be easily generated^6,41,42^ whereas NCCs of more caudal regions are not fully specified in the differentiation of hPSCs.

To address these issues, we focused on autonomic neuronal specific transcription factor *PHOX2B* and generated PHOX2B::eGFP reporter hPSC lines. Given the long-sustained *PHOX2B* expression (from progenitor cells to mature neurons), we hypothesized that tracking *PHOX2B* expression using the reporter system can help optimize the differentiation protocol. In the present study, we found the combination of PHOX2B::eGFP reporter hPSC lines and the migratory NCC marker CD49d enabled us to visualize *PHOX2B*-expressing NCCs in a heterogeneous pool of differentiating cells. In addition, modulation of rostro-caudal and dorso-ventral axis specification allowed us to generate sympathetic neuronal lineage cells from hPSCs.

BMPs are essential for differentiation toward sympathetic lineages *in vitro* and *in vivo*^23,33^. In addition, sympathetic neuronal progenitor cells from mouse embryonic sympathetic ganglia can differentiate into neurons *in vitro* in neurosphere cultures^31,32^. We combined these two ideas and successfully induced highly purified SNs from *PHOX2B*-expressing NCCs while expanding the number of cells. Furthermore, this neurosphere culture method was suitable for the selective expansion of sympathetic neuronal lineage cells. Indeed, we were able to robustly generate SNs from four different hPSC lines without cell sorting. This may have been possible because the culture media contained BMP4, thus resulting in some selection bias regarding sphere formation and propagation.

In our differentiation culture, we did not detect chromaffin cells (ChCs), which express PHOX2B, TH, DBH and Chromogranin A and lack axonal morphology and Peripherin expression^10^. We supposed that ChCs are derived from a specific level of the rostro-caudal axis (caudal thoracic region), and they should be differentiated through distinct signals^36^. In addition to this, SNs and ChCs do not share progenitor cells, and multipotent peripheral glial progenitor cells give rise to ChCs^43^. We suppose that our differentiation protocol did not produce ChCs and their progenitor cells because of the strong bias for deriving SN-lineage cells.

PHOX2B^+^SOX10^+^ mouse embryonic sympathetic ganglion progenitors show bipotency for neurons and glial cells^30–32^. However, using our differentiation protocol, it is difficult to detect a distinct glial cell population (SOX10^+^GFAP^+^ or SOX10^+^S100β^+^) even in the later stage of the differentiation (days 60–90, data not shown). The efficiency to glial commitment seemed extremely low, because PHOX2B^−^SOX10^+^ glial progenitor cells were rare in neurosphere culture with BMP4. While we do not exclude the possibility that gliogenesis is subsequent to neurogenesis, the high efficiency suggests our differentiation protocol is optimized to derive SNs over sympathetic glial cells.

Although our system successfully generated hPSC-derived neurons, the functionality of the cells has yet to be established. In this report, we emphasize the validity of tracking *PHOX2B* expression, which enabled us to refine the differentiation protocol towards sympathetic neuronal lineage cells. For future works, functional assays including electrophysiological assessments and modeling functional coupling with other types of cells such as cardiomyocytes is recommended^8^.

The ANS is a control system that regulates bodily integrated functions. Autonomic dysregulation, which occurs with congenital or acquired autonomic neuropathies^44,45^, may affect patients’ long-term quality of life. Like for other subtypes of neurons, hPSCs are useful as a source of autonomic neurons. Given that our differentiation system mimics the natural course of development of SNs, this protocol may be a useful tool for *in vitro* studies of sympathetic neuronal development. In addition, our approach will contribute to disease modeling and the discovery of new drugs for treating various kinds of sympathetic neuropathies.

## Experimental Procedures

### Study approval

The use of human ESCs was approved by the Ministry of Education, Culture, Sports, Science and Technology of Japan (MEXT). The study plan for recombinant DNA research was approved by the recombinant DNA experiments safety committee of Kyoto University. All methods were performed in accordance with the relevant guidelines and regulations. Written informed consent was obtained from the donors of the cells.

### Cell lines

The hESC lines KhES1 and KhES3^46^ were kindly provided by Dr. Hirofumi Suemori (Institute for Frontier Medical Sciences, Kyoto University, Kyoto, Japan). The human iPSC lines 409B2^47^ and 604A1^48^ were kindly provided by Dr. Shinya Yamanaka (Center for iPS Cell Research Application, Kyoto University, Kyoto, Japan). These cell lines were maintained on Growth Factor Reduced Matrigel Matrix (Corning)-coated cell culture plates with mTeSR1 medium (STEMCELL TECHNOLOGIES), as described previously^49^.

### Plasmid construction

For TALEN plasmid construction, TALEN repeat variable di-residues (RVDs) were designed using the TAL Effector Nucleotide Targeter 2.0 (https://tale-nt.cac.cornell.edu/). TALEN-encoding plasmids were assembled using the Golden Gate TALEN and TAL Effector Kit 2.0 and its protocol for assembly of the TALEN-encoding plasmids^50^ (Addgene). The mammalian expression vector with modified FokI was a kind gift from Dr. Takashi Yamamoto of Hiroshima University. All information on the TAL effector sequences and binding sites are listed in Supplementary Table 1.

For targeting vector construction, 1 kbp of PCR-amplified homology arms was cloned into the 3′ side of a loxP-neo-loxP cassette vector. Then, the 1 kbp 5′ homology arm (PCR amplified), T2A peptide sequence (annealed oligonucleotide pair) and eGFP open reading frame (ORF; without first ATG; PCR amplified) were cloned into the 5′ side of the loxP-neo-loxP cassette vector using the In-Fusion HD cloning kit (Clontech) in a seamless manner. PrimeSTAR GXL DNA Polymerase (TaKaRa) was used for PCR amplification, and all DNA fragments amplified by PCR were completely sequenced after cloning. The primers and oligonucleotides used for plasmid construction are listed in Supplementary Table 2.

For Cre-expressing vector construction, Cre with a nuclear localization signal (*NLS*-*Cre*) ORF was cloned into the multi-cloning site of a pLV-EF1a-MCS-IRES-RFP-Puro vector (BiOSETTIA).

### Transfection and stable line generation

For TALEN genome editing, transfection was performed using NEPA21 Super Electroporator (NEPAGENE) in accordance with the manufacturer’s instructions. Briefly, cells were dissociated with StemPro Accutase Cell Dissociation Reagent (Gibco) into single cells. Then, 1 million cells were transfected with 2 µg of each TALEN plasmid and 6 µg of the targeting vector plasmid in 1 cuvette and quickly reseeded onto Growth Factor Reduced Matrigel Matrix-coated 6-cm cell culture dishes (BD Falcon) with mTeSR1 medium supplemented with 50 µM Y27632 (Merck Millipore). Y27632 (10 µM) was used 24 to 48 h after transfection to promote cell survival. Cells were selected with 100 mg/ml G418 (Wako) starting 3 days after transfection. The surviving clones were isolated 10–14 days after drug selection and expanded for further experiments.

For Cre-loxP deletion, hPSCs were passaged as usual in 3.5-cm dishes (BD Falcon). Two days later, 3 µg of Cre-expression plasmid was transfected using FuGene HD Transfection Reagent (Promega) in accordance with the manufacturer’s instructions. Cells were selected with 500 ng/ml puromycin (InvioGen) starting 2 days after transfection. Four days after transfection, the surviving cells were dissociated and passaged onto mitotically inactivated SNL feeder cells with Primate ES cell medium (ReproCELL) supplemented with 5 ng/mL FGF2 (Wako) and 10 µM Y27632. The next day, Y27632 was withdrawn. Fourteen days after passage, single colonies were isolated and expanded for further experiments.

### Genomic PCR

Genomic DNA was extracted using QIAamp DNA Blood Mini Kit (QIAGEN) in accordance with the manufacturer’s instructions. Genomic PCR for detecting genomic integration at the target site was performed with PrimSTAR GXL DNA Polymerase. The primers used are listed in Supplementary Table 2.

### Differentiation of hPSCs

For NMP-like cell induction, maintained hPSCs were dissociated into single cells using StemPro Accutase Cell Dissociation Reagent and quickly re-aggregated (10,000 cells/well) in 100 µL of Essential 6 Medium (Gibco) supplemented with 10 µM SB431542 (Sigma Aldrich), various concentrations of CHIR99021 (Merck Millipore) and 10 µM Y27632 using 96-well Ultra-Low Attachment Surface multiwall plates (Corning). On day 1, 50 µL of Essential 6 Medium supplemented with 10 µM SB431542 and CHIR99031 at the same concentration as at day 0 was added to each well of the 96-well plates.

For dorso-ventral modification, 1.5, 2.0 or 3.0 µM CHIR99021-treated day 3 aggregates were cultured in Essential 6 Medium supplemented with 20 ng/mL FGF2. Retinoic acid (RA, 0, 10, 100,1000 nM; all-trans, Sigma Aldrich), BMP4 (50 ng/mL; R&D Systems) and 1 µM Purmorphamine (Tocris Bioscience) were added as indicated in Fig. 1b. The medium was changed every other day until day 10.

For cranial motor neuron differentiation, 1.5 µM CHIR99021-treated day 3 aggregates were cultured in Essential 6 Medium supplemented with 20 ng/mL FGF2, 1 µM RA and 1 µM Purumorphamine until day 10. The medium was changed every other day. At day 10, the cells were dissociated into single cells using StemPro Accutase Cell Dissociation Reagent, and CD49d^−^eGFP^+^ cells were sorted by fluorescence-activated cell sorting (FACS, see below). The sorted cells (100,000 cells/cm^2^) were cultured on Growth Factor Reduced Matrigel Matrix-coated culture plates with Neurobasal Medium (Gibco) supplemented with 1x Glutamaxl (Gibco), N2 and B27 supplement (Gibco), 100 nM Compound E (Abcam), 10 ng/mL BDNF (R&D Systems) and 10 ng/mL GDNF (R&D Systems). 20 µM Y27632 was added during the first two days after sorting. The medium was changed every other day, and the cells were passaged once at 7–10 days after sorting on Growth Factor Reduced Matrigel Matrix-coated culture plates or glass-bottom dishes (MATSUNAMI).

For the differentiation of sympathetic NCCs and SNs, 2.0 µM CHIR99021-treated day 3 aggregates were cultured in Essential 6 Medium supplemented with 20 ng/mL FGF2, 100 nM RA and 50 ng/mL BMP4 until day 10. The medium was changed every other day. On day 10, the cell aggregates were dissociated into single cells using StemPro Accutase Cell Dissociation Reagent, and CD49d^+^eGFP^+^ cells were sorted by FACS. The sorted cells were cultured in Ultra-Low Attachment Surface dishes (10 cm) or multiwall plates (6 well) (Corning) in Neurobasal Medium supplemented with 1x Glutamaxl (Gibco), N2 and B27 supplement, 20 ng/mL FGF2, 20 ng/mL EGF (R&D Systems), 50 ng/mL BMP4 and 2 µg/mL heparin (Sigma Aldrich) at a density of 100,000 cells/mL (10 cm dish; 1.2 million cells in 12 mL medium, 6 well plate; 200,000 cells in 2 mL medium). For the differentiation without cell sorting, dissociated day 10 aggregate cells were directly seeded on Ultra-Low Attachment Surface dishes (10 cm) at a density of 250,000 cells/mL (10 cm dish; 4 million cells in 12 mL medium). The medium was changed every 3 to 4 days, and spheres were passaged every 7 days by dissociating cells using 0.05% trypsin and 10 µg/mL DNaseI (STEMCELL TECHNOLOGIES), followed by gentle pipetting.

For neuronal maturation, spheres were transferred onto Ultra-Low Attachment Surface dishes or multiwall plates with Neurobasal Medium supplemented with 1x Glutamaxl (Gibco), N2 and B27 supplement, and the NFs NGF (R&D Systems), BDNF and GDNF (10 ng/ml each). The medium was changed every 3–4 days. After 14 days of NF treatment, the spheres were dissociated by 0.05% trypsin and 10 µg/mL DNaseI, followed by gentle pipetting. The dissociated cells were plated onto Growth Factor Reduced Matrigel Matrix-coated culture plates or glass-bottom dishes at a density of 100,000 cells/cm^2^ in DMEM (Nacalai Tesque) supplemented with 10% (v/v) FBS (Hyclone), 1x Glutamaxl (Gibco), NFs (10 ng/mL each) and 20 µM Y27632. Thereafter, the cultures were fed every two days by changing half of the medium without Y27632.

Images of cultured cells were obtained using a BZ-X710 microscope (KEYENCE).

### FCM analyses and FACS

PE conjugated mouse anti-CD49d antibody (BioLegend), Alexa Fluor 488 rat anti-GFP antibody (BioLegend) and Alexa Fluor 647 mouse anti-class III beta tubulin antibody (BD Bioscience) were used in accordance with the manufacturer’s protocol. FCM analyses were performed using a MACSQuant Analyzer 10 (Miltenyi Biotech). FACS was performed by BD FACSAria II (BD Bioscience). In all experiments using antibodies, isotype controls were used as control populations. As negative controls for eGFP fluorescence, time-matched, parental hPSC-derived cells were used.

### RNA isolation and RT-PCR

Total RNA extraction from cells was performed using RNeasy Mini kit (QIAGEN). Total RNA (1 µg) was used for reverse transcription with PrimeScript RT Master Mix (TaKaRa). RT-PCR was performed with Ex Taq Hot Start version (TaKaRa) or PrimeStar GXL DNA Polymerase (TaKaRa). The primer sets used for the RT-PCR assay are described in Supplementary Table 3.

### Immunocytochemistry and microscopy

Cells were dissociated into single cells, and seeded on glass-bottom dishes 6–12 hours prior to fixation. For immunocytochemistry for KI-67, cells were seeded on 48-well plates. Cells were fixed for 20 minutes at room temperature in 4% paraformaldehyde and permeabilized for 10 minutes at room temperature in 0.2% TritonX-100. The cells were then incubated with Block Ace (DS PHARMA BIOMEDICAL) to prevent any non-specific binding before incubation with primary antibodies for 12 h at 4 °C or for 2 h at room temperature. Secondary antibody incubations were performed for 1 h with the appropriate species-specific antiserum coupled to either FITC, Alexa647, Cy-3 (Jackson ImmunoResearch; 1/200) or Alexa555 (Invitrogen; 1/1000). After staining nuclei with DAPI (Sigma Aldrich; 1/1000), cells were imaged using an FV1000 or FV10i confocal microscope (Olympus). To acquire KI-67 immunocytochemistry images, cells were imaged using a BZ-X710 microscope (KEYENCE). All antibodies were diluted in Block Ace. The following primary antibodies were used at the indicated concentrations: chicken anti-GFP (Abcam; 1/5000), goat anti-PHOX2B (Santa Cruz; 1/200), goat anti-ChAT (Millipore; 1/200), mouse anti-TUBBIII (Biolegend; 1/1000), rabbit anti-SOX10 (Abcam; 1/200), mouse anti-HOXB7 (R&D Systems; 1/50), rabbit anti-TH (Millipore; 1/1000), rabbit anti-DBH (Immunostar; 1/400), goat anti-PRPH (Santa Cruz; 1/200) and mouse anti-alpha smooth muscle actin (Abcam; 1:400).

### Quantification of the results of the immunocytochemical analyses

Samples were imaged under identical gain and exposure settings. To calculate the average number of DAPI^+^, PHOX2B^+^, SOX10^+^, eGFP^+^, HOXB7^+^ or TH^+^ cells, three to nine visual fields per preparation were counted using the ImageJ software program in an automated manner. To calculate the average number of ChAT^+^, PRPH^+^ or DBH^+^ cells, three to nine visual fields per preparation were counted manually.

### Quantification of NA

The concentration of NA in the culture supernatant was measured as previously reported^8^. Cultured SNs (on 12 well plates) were incubated with 1 mL of HBSS (Gibco) for 15 min. The media was collected as a control. The cells were incubated with 50 mM KCl in HBSS (total 1 mL) for another 15 min, and then the media was collected. Following media collection, the media samples were centrifuged at 300 *g* for 5 minutes to eliminate cells or debris. To prevent NA degradation, 1 mM EDTA (Gibco) and 4 mM sodium metabisulfite (Nacalai Tasque) were added to the samples, which were stored at −80 °C until the analysis. The total NA levels of samples (300 µL) were quantified using Epinephrine/Norepinephrine Elisa kit (Abnova) in accordance with the manufacturer’s instruction except for the minimum concentration of the standard samples (1 ng/mL, successful for drawing standard curves, data not shown).

### Statistical analyses

Microsoft Excel 2013 software was used for the statistical analyses. The results are expressed as the mean ± standard division (s.d.). Statistical significance was determined using Student’s *t*-test. ‘n’ represents the number of independent differentiations.

### Full length gel images

Full length gel images for electrophoresis were shown in Supplementary Figures 5–7.

## Electronic supplementary material

Dataset 1
