# Supplementary material for: Efficient derivation of sympathetic neurons from human pluripotent stem cells with a defined condition
Source: Sci Rep. 2018 Aug 27;8:12865. doi: 10.1038/s41598-018-31256-1 (PMC6110806; doi:10.1038/s41598-018-31256-1)
Supplement: Supplementary file 1 — Dataset 1 [file 41598_2018_31256_MOESM1_ESM.pdf]

Supplementary information for,

Efficient derivation of sympathetic neurons from human pluripotent stem cells with a defined condition

Authors

Kosuke Kirino, Tatsutoshi Nakahata, Tomoaki Taguchi and Megumu K. Saito

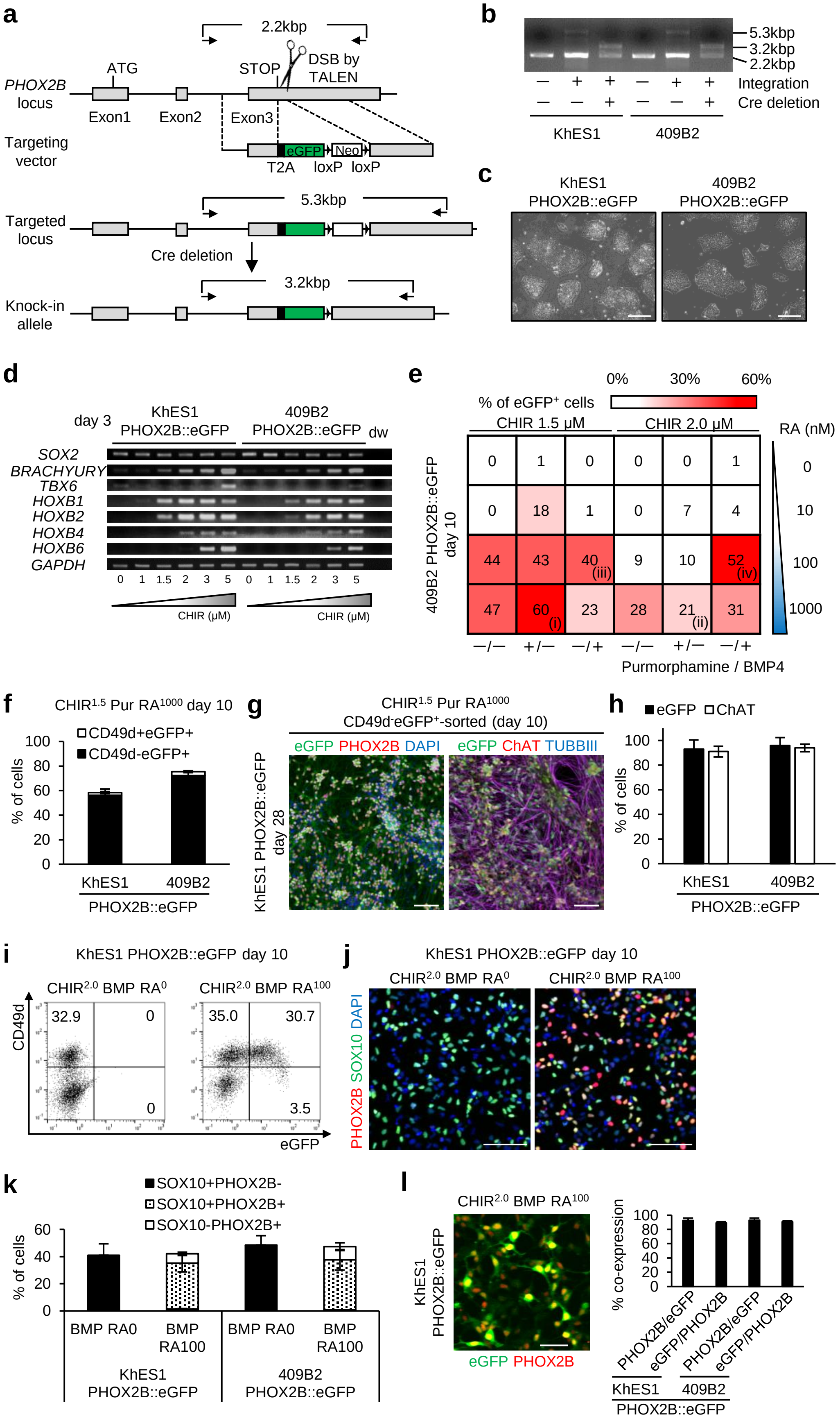
a
2.2kbp
DSB by TALEN
ATG
STOP
PHOX2B locus
Exon1
Exon2
Exon3
Targeting vector
eGFP
Neo
T2A
loxP
loxP
5.3kbp
Targeted locus
Cre deletion
3.2kbp
Knock-in allele
b
5.3kbp
3.2kbp
2.2kbp
Integration
Cre deletion
KhES1
409B2
c
KhES1 PHOX2B::eGFP
409B2 PHOX2B::eGFP
d
day 3
KhES1 PHOX2B::eGFP
409B2 PHOX2B::eGFP
dw
SOX2
BRACHYURY
TBX6
HOXB1
HOXB2
HOXB4
HOXB6
GAPDH
0 1 1.5 2 3 5 0 1 1.5 2 3 5
CHIR (μM)
CHIR (μM)
e
0% 30% 60%
% of eGFP+ cells
CHIR 1.5 μM
CHIR 2.0 μM
RA (nM)
409B2 PHOX2B::eGFP day 10
0 1 0 0 0 1
0 18 1 0 7 4
44 43 40 (iii) 9 10 52 (iv)
47 60 (i) 23 28 21 (ii) 31
0
10
100
1000
−/− +/− −/+ −/− +/− −/+
Purmorphamine / BMP4
f
CHIR1.5 Pur RA1000 day 10
CD49d+eGFP+
CD49d-eGFP+
% of cells
100
80
60
40
20
0
KhES1
409B2
PHOX2B::eGFP
g
CHIR1.5 Pur RA1000
CD49d-eGFP+-sorted (day 10)
eGFP PHOX2B DAPI
eGFP ChAT TUBBIII
KhES1 PHOX2B::eGFP day 28
h
eGFP
ChAT
% of cells
KhES1
409B2
PHOX2B::eGFP
i
KhES1 PHOX2B::eGFP day 10
CHIR2.0 BMP RA0
CHIR2.0 BMP RA100
CD49d
32.9
0
0
35.0
30.7
3.5
eGFP
j
KhES1 PHOX2B::eGFP day 10
CHIR2.0 BMP RA0
CHIR2.0 BMP RA100
PHOX2B SOX10 DAPI
k
SOX10+PHOX2B-
SOX10+PHOX2B+
SOX10-PHOX2B+
% of cells
60
40
20
0
BMP RA0
BMP RA100
BMP RA0
BMP RA100
KhES1 PHOX2B::eGFP
409B2 PHOX2B::eGFP
l
CHIR2.0 BMP RA100
KhES1 PHOX2B::eGFP
eGFP PHOX2B
% co-expression
100
80
60
40
20
0
PHOX2B/eGFP
eGFP/PHOX2B
PHOX2B/eGFP
eGFP/PHOX2B
KhES1
409B2
PHOX2B::eGFP

Supplementary Figure 1. Detection of *PHOX2B*-expressing cells using PHOX2B::eGFP knocked-in hPSC clones, related to Figure 1.

(a) A schematic illustration of *PHOX2B* gene targeting using TALEN-mediated genome editing. **(b)** Genomic PCR showing targeted integration at the *PHOX2B* 3'UTR region. **(c)** Representative images of PHOX2B::eGFP knocked-in hPSC lines (scale bars = 200 µm). **(d)** RT-PCR analyses of *SOX2*, *BRACHYURY*, *TBX6*, *HOXB1*, *HOXB2*, *HOXB4* and *HOXB6* in day 3 aggregates at various concentrations of CHIR99021. **(e)** A heat map image showing the percentage of $eGFP^{+}$ cells on day 10 of differentiation using 409B2 PHOX2B::eGFP under various conditions. **(f)** Quantification of $eGFP^{+}$ and $CD49d^{+\ or\ -}$ cells on day 10 under $CHIR^{1.5\mu M}Pur^{+}RA^{1000nM}$ conditions with FCM analyses (mean ± s.d., n = 3). **(g)** Immunocytochemistry analyses for eGFP, PHOX2B, ChAT and TUBBIII in $eGFP^{+}CD49d^{-}$ cell-derived neurons (day 28, scale bars = 50 µm). **(h)** Quantification of $eGFP^{+}$ and $ChAT^{+}$ cells in **(g)** (mean ± s.d., n = 3). **(i)** Representative FCM plots of day 10 KhES1 PHOX2B::eGFP-derived aggregates under $CHIR^{2.0\mu M}BMP^{+}RA^{0nM}$ and $CHIR^{2.0\mu M}BMP^{+}RA^{100nM}$ conditions. **(j)** Immunocytochemistry analyses for PHOX2B and SOX10 on day 10 of differentiation under $CHIR^{2.0\mu M}BMP^{+}RA^{0nM}$ and $CHIR^{2.\ 0\mu M}BMP^{+}RA^{100nM}$ conditions (scale bars = 50 µm). **(k)** Quantification of $SOX10^{+}$ and $PHOX2B^{+}$ cells in **(j)** (mean ± s.d., n = 3). **(l)** Quantification of $PHOX2B^{+}$ cells in $eGFP^{+}$ cells (PHOX2B/eGFP) and of $eGFP^{+}$ cells in $PHOX2B^{+}$ cells (eGFP/PHOX2B). The left image shows immunocytochemistry findings for eGFP and PHOX2B on day 10 under $CHIR^{2.0\mu M}BMP^{+}RA^{100nM}$ condition (scale bar = 50 µm). The right graph shows the quantitative data (mean ± s.d., n = 3).

DSB = double-strand break, CHIR = CHIR 99021, RA = retinoic acid, Pur = Purmorphamine, BMP = BMP4.

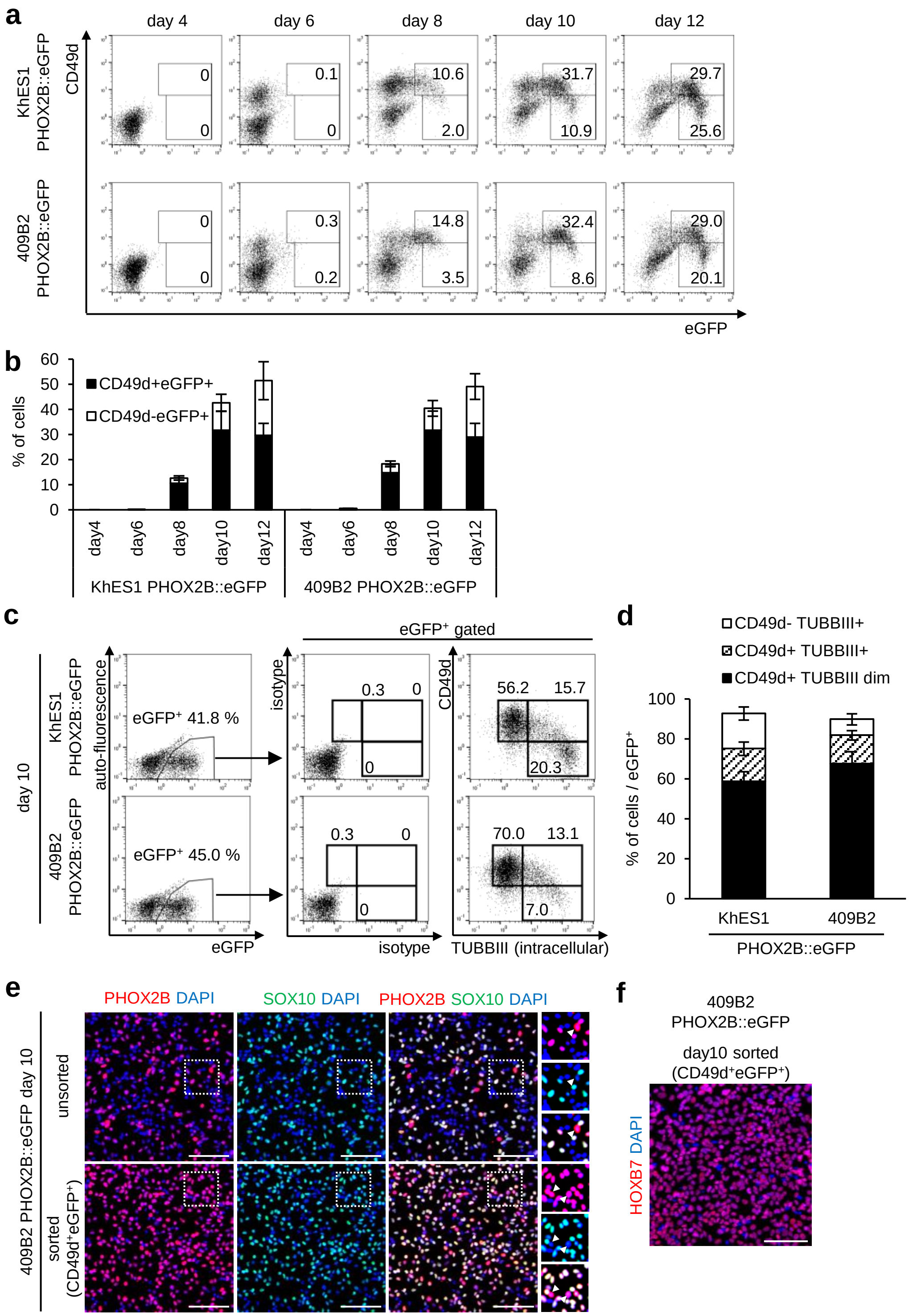

Supplementary Figure 2. Characterization of cells among aggregates under CHIR$^{2.0\mu M}$BMP4^{+}RA^{100nM} conditions, related to Figure 2.

(a) Representative FCM plots of time course analyses of eGFP and CD49 expression. **(b)** Quantification of GFP^{+} and CD49d^{+} cells in the time course analyses in **(a)** (mean ± s.d., n = 3). **(c)** Representative FCM plots for eGFP, CD49d and TUBBIII in day 10 aggregates. Note that intracellular eGFP and TUBBIII were stained after extracellular staining of CD49d, fixation and permeabilization. **(d)** Quantification of CD49d^{+}, TUBBIII dim and TUBBIII^{+} cells in the FCM analyses in **(c)** (mean ± s.d., n = 3). **(e)** Immunocytochemistry analyses of unsorted and CD49d^{+}eGFP^{+}-sorted day 10 differentiated cells for PHOX2B and SOX10 (409B2 PHOX2B::eGFP, scale bars = 50 µm). The white dashed boxes mark the areas enlarged in the right panels. The white arrowheads indicate PHOX2B^{+}SOX10^{-} cells. **(f)** An immunocytochemistry analysis for HOXB7 on day 10 (CD49d^{+}eGFP^{+}-sorted) of differentiation (409B2 PHOX2B::eGFP, scale bars = 50 µm).

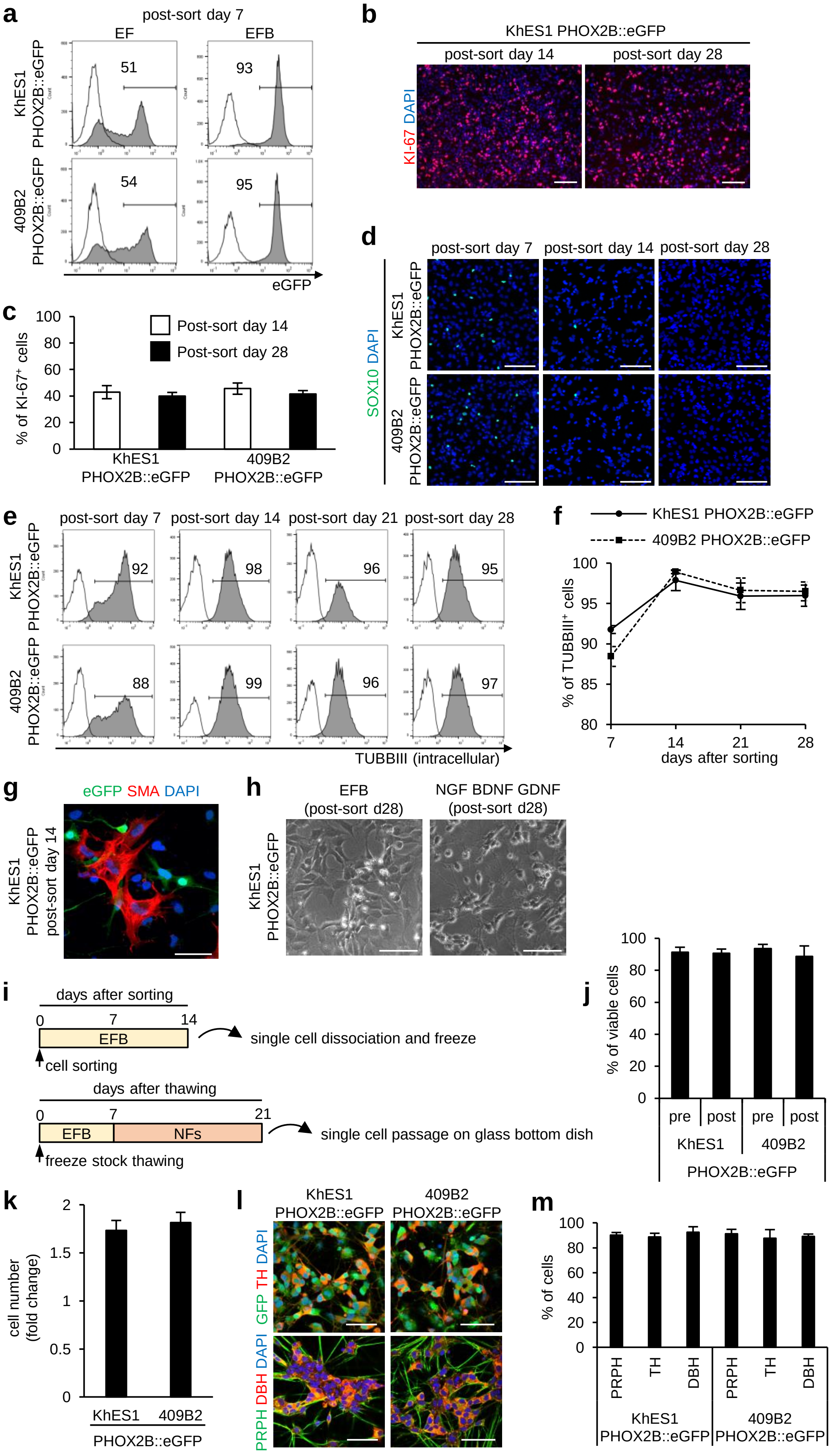
a
post-sort day 7
EF
EFB
KhES1 PHOX2B::eGFP
409B2 PHOX2B::eGFP
51
93
54
95
eGFP
b
KhES1 PHOX2B::eGFP
post-sort day 14
post-sort day 28
KI-67 DAPI
c
% of KI-67+ cells
Post-sort day 14
Post-sort day 28
KhES1 PHOX2B::eGFP
409B2 PHOX2B::eGFP
d
post-sort day 7
post-sort day 14
post-sort day 28
SOX10 DAPI
e
post-sort day 7
post-sort day 14
post-sort day 21
post-sort day 28
92
98
96
95
88
99
96
97
TUBBIII (intracellular)
f
KhES1 PHOX2B::eGFP
409B2 PHOX2B::eGFP
% of TUBBIII+ cells
days after sorting
g
eGFP SMA DAPI
KhES1 PHOX2B::eGFP post-sort day 14
h
EFB (post-sort d28)
NGF BDNF GDNF (post-sort d28)
i
days after sorting
EFB
cell sorting
single cell dissociation and freeze
days after thawing
EFB
NFs
freeze stock thawing
single cell passage on glass bottom dish
j
% of viable cells
pre
post
KhES1
409B2
PHOX2B::eGFP
k
cell number (fold change)
l
GFP TH DAPI
PRPH DBH DAPI
m
% of cells
PRPH
TH
DBH

Supplementary Figure 3. Optimized neurosphere culture is suitable for the induction of sympathetic neurons from sorted CD49d$^+$eGFP$^+$ cells, related to Figure 3.

(a) An FCM analysis of the eGFP expression of post-sorting day 7 neurosphere cells with or without BMP4 (gray). Parental clones without PHOX2B::eGFP reporter (white) were used as negative control. **(b)** Immunocytochemistry analyses for KiI-67 on post-sorting days 14 and 28. **(c)** Quantification of KI-67$^+$ cells in **(b)** (mean ± s.d., n = 3). **(d)** Immunocytochemistry analyses for SOX10 on post-sorting days 7, 14 and 28. **(e)** FCM analyses of the TUBBIII expression in post-sorting days 7, 14, 21 and 28 neurosphere cells (gray). Isotype controls were used to determine the negative population (white). **(f)** Quantification of TUBBIII$^+$ cells in the FCM analyses in **(e)** (mean ± s.d., n = 3). **(g)** An Immunocytochemistry analysis for eGFP and SMA in post-sorting day 14 cells in adherent culture with BMP4. **(h)** Morphology of neurosphere cells with or without treatment of NFs (scale bars = 100 μm). **(i)** A diagram of the stock and culture after thawing the neurosphere cells. **(j)** Cell viabilities of neurosphere cells based on trypan blue staining just before preparing freeze stocks (pre) and just after thawing stocks (post) (mean ± s.d., n = 3). **(k)** Numbers of neurosphere cells 7 days after thawing. Values show the fold-change relative to the number of cells on the day of thawing (mean ± s.d., n = 3). **(l)** Immunocytochemistry analyses for eGFP, TH, PRPH and DBH of stocked neurosphere cells after neuronal induction culture (scale bars = 50 μm). **(m)** Quantification of PRPH$^+$, TH$^+$ and DBH$^+$ cells in **(l)** (mean ± s.d., n = 3).

EF = EGF + FGF2, EFB = EGF + FGF2 + BMP4, SMA = smooth muscle actin, NFs = neurotrophic factors: NGF, BDNF and GDNF.

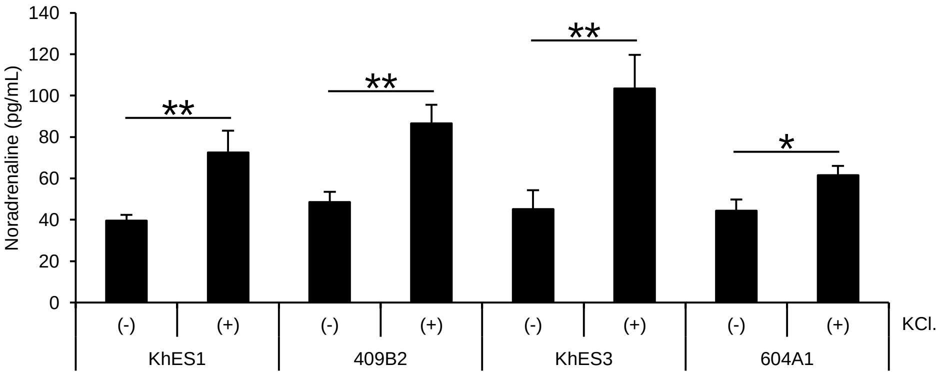

Supplementary Figure 4. Noradrenaline release of reporter-free hPSC-derived SN-like cells, related to Figure 4.

Quantification of the noradrenaline concentrations of the supernatant of cultured SNs derived from reporter-free hPSCs on day 32 with (+) or without (-) KCl treatment (mean ± s.d., n = 3, *$P < 0.05$, **$P < 0.01$; Student's *t*-test).

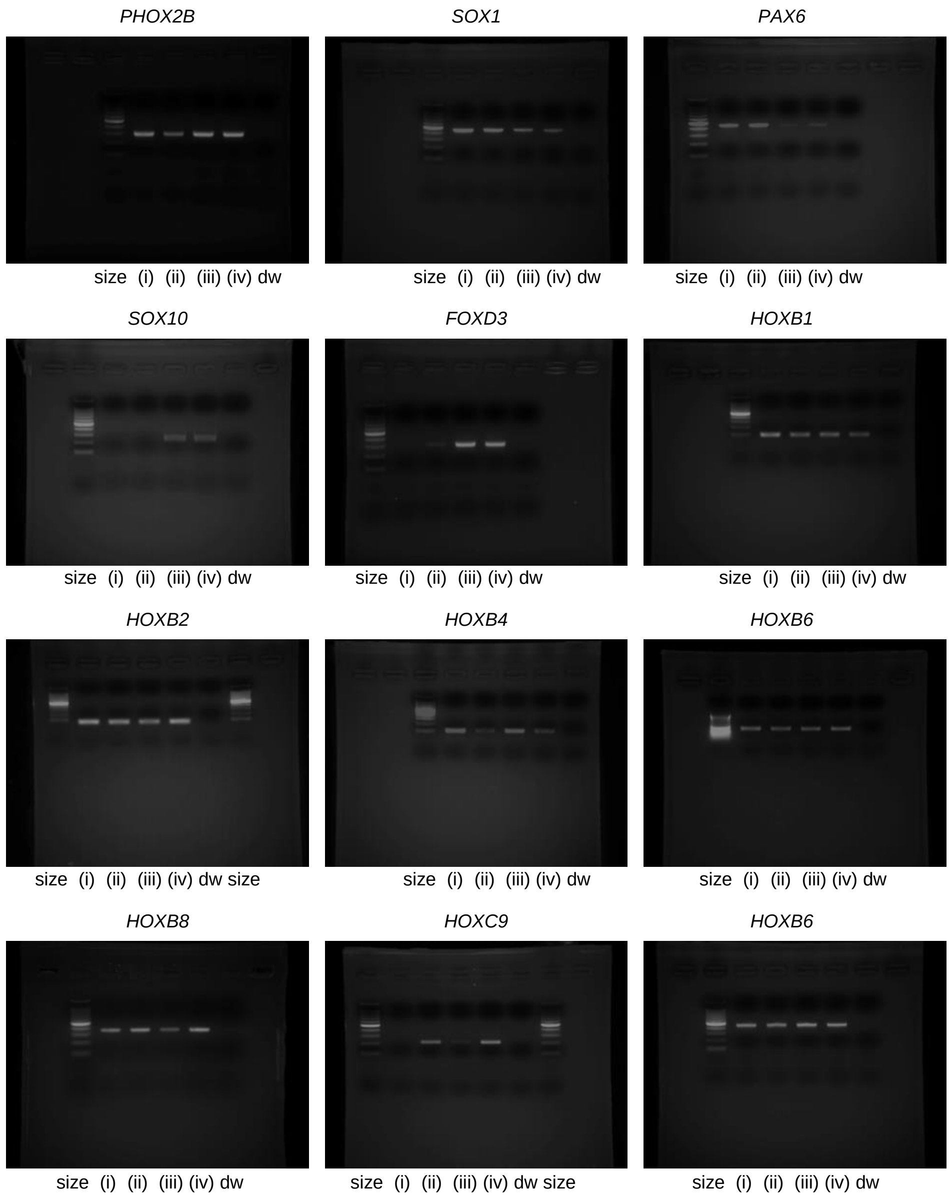

Supplementary Figure 5. Full-length gel images for Figure 1c.
(i) – (iv) indicates the conditions as follows: (i) CHIR$^{1.5\mu M}$ Pur^{+} RA^{1000nM} , (ii) CHIR$^{2.0\mu M}$ Pur^{+} RA^{1000nM}, (iii) CHIR$^{1.5\mu M}$ BMP^{+} RA^{100nM} and (iv) CHIR$^{2.0\mu M}$ BMP^{+} RA^{1000nM}.

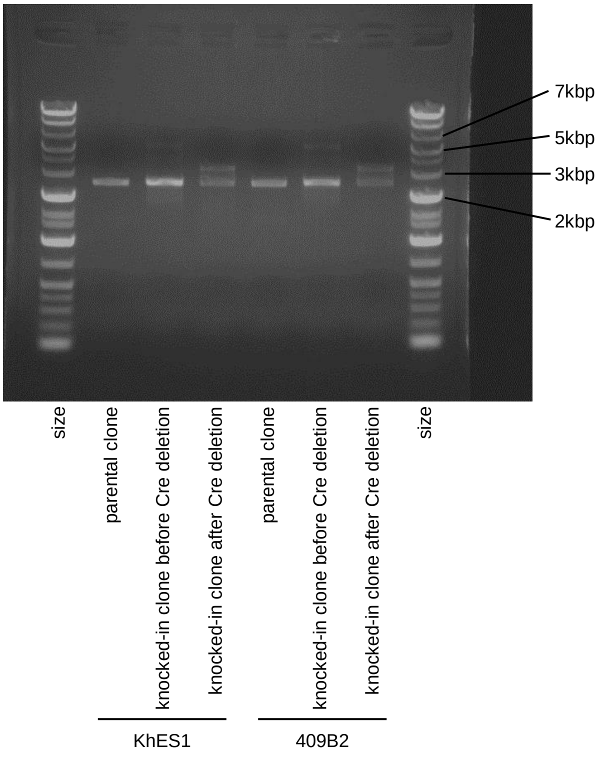

Supplementary Figure 6. Full-length gel images for Supplementary Figure 1b.

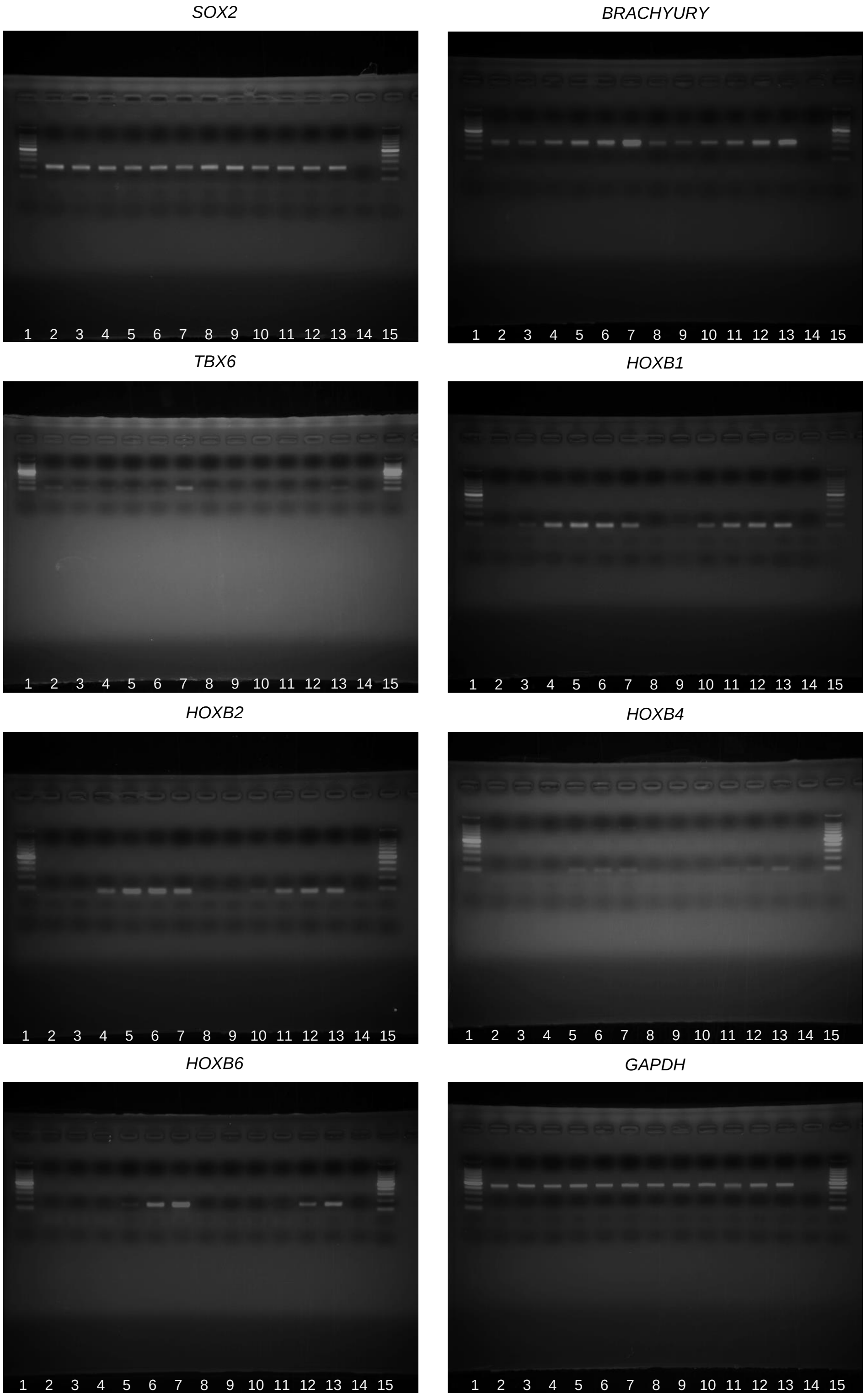

1 and 15; size marker
2; 0 µM CHIR99021
3; 1 µM CHIR99021
4; 1.5 µM CHIR99021
5; 2 µM CHIR99021
6; 3 µM CHIR99021
7; 5 µM CHIR99021
(2–7: KhES1 PHOX2B::eGFP)

8; 0 µM CHIR99021
9; 1 µM CHIR99021
10; 1.5 µM CHIR99021
11; 2 µM CHIR99021
12; 3 µM CHIR99021
13; 5 µM CHIR99021
(8–13: 409B2 PHOX2B::eGFP)
14; distilled water

Supplementary Figure 7. Full-length gel images for Supplementary Figure 1d.

Supplementary Table 1. Custom TALENs and target sites.

Gene	TAL Plasmid	Repeat number TAL left , TAL right	Spacer	TAL left, TAL right RVD Sequences	DNA Target Sequence with Spacer (TAL left, TAL right underlined)
PHOX2B	pCSGGS-TAL-NC2	16, 16	15	TAL left; NG HD NG NN NN NI NI NG HD HD NG NN HD NN NN HD<br>TAL right; NG NN NN HD NG HD NN HD HD HD NN HD NG NN NG HD	5’ -TCTGGAATCCTGCGGC GGCGGCGGCGGCGGC GACAGCGGGCGAGCCA- 3’

Supplementary Table 2. Primers and oligonucleotides used for plasmid construction and genotyping.

name	sequence (5'-3')	usage
IF-TALPHOX2B5'arm-F	ACCGCGGTGGCGGCCCGGAAACTGTGACTTGCATC	PCR for cloning
IF-TALPHOX2B5'arm-R	TCTGCCCTCGAACATACTGCTCTTCACTAAGGCGG	
oligo-6bp-T2Apeptide-F	ATGTTCGAGGGCAGAGGAAGTCTTCTAACATGCGGTGACGTGGAGGAGAATCCCGGCCCT	annealing for cloning
oligo-6bp-T2Apeptide-R	AGGGCCGGGATTCTCCTCCACGTCACCGCATGTTAGAAGACTTCCTCTGCCCTCGAACAT	
IF-eGFP-F	GAGAATCCCGGCCCTGTGAGCAAGGGCGAGG	PCR for cloning
IF-eGFP-R	GCAGCCCGGGGGATCTTACTTGTACAGCTCGTCCATG	
IF-TALPHOX3'arm-F	AAATGTCGACCTCGAGTAGGCCCAAGGCTATTGTCG	PCR for cloning
IF-TALPHOX3'arm-R	TATAGGGCGAATTGGGTACCGTGTGCCTTTTCCTTGCTCG	
genotype-F	ATTTCGGGGACAGGAAGTGC	PCR for genotyping
genotype-R	AGGATGTGAGTCCAGTTCGG	

Supplementary Table 3. Primers list used for RT-PCR.

target	forward (5'-3')	reverse (5'-3')	size
GAPDH	ACCACAGTCCATGCCATCAC	TCC ACC ACCCTG TTG CTG TA	452
PHOX2B	GGCTGAGCCATCCAGAACCT	GTCCGTGAAGAGTTTGTAAG	289
SOX1	CAATGCGGGGAGGAGAAGTC	CTCTGGACCAAACTGTGGCG	464
PAX6	GGCAACCTACGCAAGATGGC	TGAGGGCTGTGTCTGTTCGG	459
SOX10	ATACGACACTGTCCCGGCCCTAAA	TTCTCCTCTGTCCAGCCTGTTCTC	247
FOXD3	CCATTCTTAGCGTGCCACTC	GTTTGCTCGACCAGCTTAGGTG	310
HOXB1	GAGCTTTGCACCGGCCTAT	CTTCATCCAGTCGAAGGTCCG	103
HOXB2	CCTAGCCTACAGGGTTCTCTC	CACAGAGCGTACTGGTGAAAAA	79
HOXB4	AAAGAGCCCGTCGTCTACC	GTGTAGGCGGTCCGAGAG	101
HOXB6	GAACTGAGGAGCGGACTCAC	CTGGGATCAGGGAGTCTTCA	153
HOXB8	AGCCTCCTTGTGCAATTG	GTAACAATTGCCCACAGC	358
HOXC9	GGGAGGGTTCAGTGTTGAGA	GGGATGACCTGGACCAAATA	216
SOX2	TGGACAGTTACGCGCACAT	CGAGTAGGACATGCTGTAGGT	215
BRACHYURY	CTTCCCTGAGACCCAGTTCA	CAGGGTTGGGTACCTGTCAC	292
TBX6	AAGTACCAACCCCGCATACA	TAGGCTGTCACGGAGATGAA	113
